# mSphere of Influence: Considering Complex Mutational Processes That Shape Microbial Virulence

**DOI:** 10.1128/mSphere.00578-19

**Published:** 2019-08-21

**Authors:** Matthew Zack Anderson

**Affiliations:** a Department of Microbiology, The Ohio State University, Columbus, Ohio, USA; b Department of Microbial Infection and Immunity, The Ohio State University, Columbus, Ohio, USA

**Keywords:** *Candida albicans*, adaptation, loss of heterozygosity, ploidy, trisomy

## Abstract

Matt Anderson works in the field of genetics and infectious disease, with a focus on the human fungal pathogen Candida albicans. In this mSphere of Influence article, he reflects on how two papers, “Gene Flow Contributes to Diversification of the Major Fungal Pathogen Candida albicans” (J. Ropars, C. Maufrais, D. Diogo, M. Marcet-Houben, A. Perin, et al., Nat Commun 9:2253, 2018, https://doi.org/10.1038/s41467-018-04787-4) and “Selection of Candida albicans Trisomy during Oropharyngeal Infection Results in a Commensal-Like Phenotype” (A. Forche, N. V. Solis, M. Swidergall, R. Thomas, A. Guyer, et al., PLoS Genet 15:e1008137, 2019, https://doi.org/10.1371/journal.pgen.1008137), made an impact on him by incorporating less commonly investigated mechanisms of genome evolution into the context of microbial adaptation.

## COMMENTARY

In most microbial systems lacking meiosis, evolution is considered to proceed through the iterative accumulation of site-specific mutations over time. However, the presence of linear chromosomes and diploid or higher-ploidy states provides frequent additional avenues for adapting to new or changing environments. These include transition between ploidy states and aneuploid formation, recombination among chromosomes, and loss of heterozygosity, a process in which alleles from one chromosome are lost and replaced by alleles from the remaining homolog(s). Importantly, some of these genetic changes are reversible, allowing cells in a population to explore evolutionary space without requiring a full commitment to carry that event into successive generations. Two recent papers have guided my thoughts on how these genetic mechanisms contribute to phenotypic diversity in the commensal yeast and opportunistic pathogen Candida albicans and, specifically, how they regulate disease outcomes.

The paper “Gene Flow Contributes to Diversification of the Major Fungal Pathogen Candida albicans” published by Christophe d’Enfert’s lab significantly advanced descriptions of the breadth of genetic diversity within this yeast (1). A collection of 182 C. albicans isolates were fully sequenced, most of which originated from commensal sites of colonization or superficial infections. Approximately 90% of strains were true diploids, although they harbored widespread and diverse regions of loss of heterozygosity (LOH), segments of DNA in which one of the diploid alleles has been lost and replaced by the other remaining allele. Construction of the strain phylogeny indicated that sequenced isolates evolved primarily through clonal evolution, with the gradual acquisition of iterative mutations. However, two strain clusters showed strong evidence of genetic admixture, suggesting that mating and parasexual reproduction of isolates from other clades produced these lineages. Analysis of a third cluster previously described as the Candida africana subspecies demonstrated that these strains suffered from reduced growth compared to that of other isolate clusters, likely due to a combination of frequent gene-disrupting mutations and evidence of nearly complete genome-wide LOH.

Investigation of these isolates built on prior work detailing C. albicans genetic diversity by increasing the number of sequenced strains approximately 9-fold. Unlike in previous reports, isolates from this set were almost entirely diploid. My interpretation of these results is that the prior observation of frequent aneuploidy among C. albicans clinical isolates reflected antifungal drug exposure prior to strain isolation that produced aberrant karyotypes derived from an originally euploid state. Of profound importance was the detection of recombinant isolates encoding genomic tracts identical to consensus genotypes from two different clades. The strong evidence of admixed strains within this study, despite meiosis not having been observed in C. albicans, demonstrated a clear role for the alternative mating pathway, the parasexual cycle, in shaping natural populations colonizing the host. Genetic admixture also provided a solution to the dilemma of Mueller’s ratchet, in which fixation of certain alleles through LOH ultimately lead to reduced fitness and an evolutionary dead end. However, this study also highlighted the C. africana clade as an example of Mueller’s ratchet. These strains encoded a nearly homozygous genome and displayed reduced fitness and disease potential compared to those of other C. albicans lineages. It is possible that these changes may be beneficial in additional untested niches, as they have persisted through evolutionary time and are commonly associated with genitourinary infections. Collectively, these results have challenged my conceptual framework of iterative mutation in C. albicans and refocused my interests toward using parasexual processes and large-scale LOH to dissect genetic mechanisms of phenotypic diversity displayed among strains.

Karyotypic changes altered host-pathogen responses in the paper titled “Selection of Candida albicans Trisomy during Oropharyngeal Infection Results in a Commensal-Like Phenotype” (2). This study, co-led by Anja Forche and Scott Filler, investigated the genetic basis for enhanced colonization in a murine model of oropharyngeal colonization. SC5314-derived strains encoding chromosome 6 (Chr6) trisomies were commonly isolated from the oral cavity in previously reported experiments, suggesting that the elevated copy number of Chr6 shifts the invasive, damaging profile of SC5314 to a commensal-like colonization phenotype. Strains trisomic for Chr6 displayed fungal burdens equivalent to those of their disomic counterparts in the oral cavity but elicited an attenuated immune response. More specifically, mice orally infected with trisomic Chr6ABB strains had a squelched chemokine response for neutrophil recruitment and antibody response associated with Th17-mediated immunity. Furthermore, Chr6 trisomic strains displayed reduced adherence to epithelial surfaces, enhanced killing by neutrophils, and reduced filamentation under a variety of *in vitro* conditions. A number of these traits were more prominent in strains harboring an extra copy of Chr6B than in strains harboring Chr6A, suggesting that specific alleles on Chr6 contribute to commensal-like phenotypes in SC5314 strains trisomic for Chr6.

This study brings new insight into the genetic determinants that oversee the balance between commensal and pathogen phenotypes. A couple of recent reports have highlighted the homozygosity of specific mutations that regulate the balance between C. albicans invasive disease and commensal-like phenotypes. However, once homozygosis occurs to promote commensalism, the strain is unable to easily revert the mutation and regain its original virulence. In contrast, gains and losses of additional chromosomes occur readily in C. albicans during *in vivo* growth, providing a rapid mechanism for interconversion between commensalism and pathogenesis. This led me to see chromosome instability during C. albicans growth in the oral cavity as a bet-hedging strategy in which Chr6 trisomic strains are able to persist under certain circumstances due to dampened immunological responses. Furthermore, the commensal-like phenotypes observed for mice infected with the Chr6 trisomic strains (maintained body weight, reduced phagocyte infiltration) centered not on the abundance or duration of fungal burden but on immune response and recruitment. Unexpectedly, dampened immunity was not due to decreases in epithelial damage, which has been observed in other C. albicans strains capable of establishing commensal persistence in the oral cavity but to adherence and uptake by oral epithelial cells *in vitro*. These results have led me to view commensal versus pathogen interactions between the host and C. albicans as multifactorial and not an all-or-nothing phenotype. Finally, an incomplete description of the locus or loci contributing to these phenotypes highlights deficits in current approaches to identifying candidate loci within regions of copy number changes to allow targeted manipulation. The existence of these challenges has furthered molded my interest in utilizing the natural processes of parasex and targeted LOH to define the genes that contribute to these processes, with clear applications to commensal-pathogen balances in host niches.
